# New methods to measure residues coevolution in proteins

**DOI:** 10.1186/1471-2105-12-206

**Published:** 2011-05-26

**Authors:** Hongyun Gao, Yongchao Dou, Jialiang Yang, Jun Wang

**Affiliations:** 1 School of Mathematical Sciences, Dalian University of Technology, 116024 Dalian, People's Republic of China; 2 College of Advanced Science and Technology, Dalian University of Technology, 116024 Dalian, People's Republic of China; 3 MPI-CAS Institute of Computational Biology, Chinese Academy of Sciences at Shanghai, 200031 Shanghai, People's Republic of China; 4 Scientific Computing Key Laboratory of Shanghai Universities, 200234 Shanghai, People's Republic of China; 5 Department of Mathematics, Shanghai Normal University, 200234 Shanghai, People's Republic of China

## Abstract

**Background:**

The covariation of two sites in a protein is often used as the degree of their coevolution. To quantify the covariation many methods have been developed and most of them are based on residues position-specific frequencies by using the mutual information (MI) model.

**Results:**

In the paper, we proposed several new measures to incorporate new biological constraints in quantifying the covariation. The first measure is the mutual information with the amino acid background distribution (MIB), which incorporates the amino acid background distribution into the marginal distribution of the MI model. The modification is made to remove the effect of amino acid evolutionary pressure in measuring covariation. The second measure is the mutual information of residues physicochemical properties (MIP), which is used to measure the covariation of physicochemical properties of two sites. The third measure called MIBP is proposed by applying residues physicochemical properties into the MIB model. Moreover, scores of our new measures are applied to a robust indicator *conn(k) *in finding the covariation signal of each site.

**Conclusions:**

We find that incorporating amino acid background distribution is effective in removing the effect of evolutionary pressure of amino acids. Thus the MIB measure describes more biological background information for the coevolution of residues. Besides, our analysis also reveals that the covariation of physicochemical properties is a new aspect of coevolution information.

## Background

A protein's function depends on its three-dimensional (3D) structure and interactions of residues [1]. When there is a mutation of functionally or structurally important residues, compensatory mutations may occur to preserve or restore the function or structure of the protein. Thus the knowledge of residues coevolution helps to predict protein function and guide experimental analysis. To quantify the coevolution of a protein chain, a multiple sequence alignment (MSA) of the chain and its homologous sequences are generated at first. Then the covariation of two sites is used as the degree of their coevolution.

In order to quantify the covariation of two sites in a given MSA, many computational methods have been developed in recent years. These methods can be divided into two groups: parametric methods and nonparametric ones [2-14]. The parametric methods incorporate maximum likelihood approximations [15], Bayesian probabilities [16], Phylogentic approaches [17] and so on. While nonparametric methods are more extensive and most of them are based on mutual information (MI). The MI model is taken from information theory [18], and uses the position specific distribution of two sites [19-21]. For example, [6] tested the effect of the size of MSAs and the mutation rate on two sources of background (finite sample size effects and phylogenetic influence). They also tested the performance of various normalizations of MI in enhancing the detection of coevolving sites. To integrate different biological constraints with the MI model, some methods focused on how to relate observation counts to expected distribution [22-24]. However, [25] pointed out that none of the estimates are more 'correct' than others since all the methods merely depend on assumptions. And it has been shown that the estimates of MI are more affected by these assumptions than by the actually observed data. The 'correct' conditional assumptions refer to the assumptions strictly matching known biological constraints. Another widely used nonparametric method is ELSC which applies a perturbation-based method to calculate explicitly the likelihood of evolutionary covariance in MSAs [26]. Although many biological constraints have been used in measuring covariation, the amino acid background distribution and their physicochemical properties are ignored in previous methods.

In order to improve covariation measures, two new biological constraints are introduced in the paper. The first constraint is the amino acid background distribution which indicates the evolutionary pressures of amino acids. It has been proved that if a residue is under relatively low evolutionary pressure, it will be readily replaced in the evolutionary history. Thus this residue will have relatively low frequency in an MSA and vice versa. In general, the frequency of a residue can be used as an estimate of its evolutionary pressure and is often called the background frequency of the residue [27]. A residue background distribution is called 'no pressure' if it can describe the distribution of amino acids subject to no evolutionary pressure [28]. Actually, it depicts evolutionary pressures for each residue exactly. However, it is difficult to estimate the 'no pressure' background distribution. Thus statistical background usually provides an alternative to approximate it. As suggested by several existing conservation measures, the *BLOSUM62 *background distribution [29] is used as the amino acid background distribution in the paper. By incorporating amino acid background distribution into account, we proposed a new method MI with amino acid background distribution (MIB).

The second constraint to improve covariation measures is the physicochemical properties which are important in predicting functional important residues [30-36]. In order to incorporate the physicochemical properties into the MI model, amino acids are often grouped into six disjoint groups or ten overlapping groups in previous works [37]. In the study, the classification of ten overlapping groups of amino acids is chosen since six disjoint groups has a deficiency that residues of different types are treated equally different. Then, a new method called the MI with physicochemical properties (MIP) is developed to estimate the variation of physicochemical properties of two given sites.

The third model is called MIBP method which is used to estimate the covariation of physicochemical properties by removing their background distributions. Moreover, our measures are applied to a robust indicator *conn(k) *[23] in finding covariation signal of each site.

## Methods

Since the reliability of MI values depends on that of MSA, the quality of MSA is important in measuring the covariation. In the paper, MSAs are downloaded from the PFAM [38] data base. And the number of sequences in an alignment should be greater than 125 [6]. Given an MSA, sequences in it are clustered at 90% sequence identity and the redundant sequences are removed. Moreover, the columns with more than 25% gaps are also removed as suggested by [23]. Gaps are also ignored when position specific frequencies are calculated. Moreover, these proposed methods are also tested on a recently published data set which was created by Capra and Singh (2007) [28]. MSAs in the data set with lower than 125 sequences are removed. After filtering, 496 MSAs remained and results on them can be found in the supplement material (Additional files 1, 2, 3 and 4). Throughout the paper, we use *N *to denote the number of sequences in the MSA and *c_pair (K,L) *to denote the pair of column *K *and column *L *used to calculate the MI based measures.

### The MI model with the amino acid background distribution (MIB)

An ordering is first specified to the 20 amino acids. Given an MSA, the amino acid position specific frequency of a column *K *for the *i*th residue is calculated as:

where count(*K^i^*) denotes the number of the *i*th (*i *= 1, ..., 20) residue in column *K*.

Similarly, the joint probability distribution of *c_pair (K,L) *for a residue pair consisting of the *i*th and *j*th residue is defined as:

where count(*K^i^*, *L^j ^*) denotes the number of rows, in which the residues in column *K *and *L *are the *i*th and *j*th residue, respectively.

Based on the above definitions, the classical MI [18] is calculated as:

Clearly, if *p*(*K^i ^*, *L^j ^*) = 0, then the value of the monomial  is 0. Moreover, if one column in a column-pair is fully conserved, then *M I *(*K*, *L*) = 0. As suggested by [6], we further normalize *M I *(*K*, *L*) by *H *(*K*, *L*), and define

where .

In order to account for the background distribution of amino acids, we introduce the marginal distribution, which is defined as:

where *q *is the *BLOSUM62 *amino acid background distribution. Then a new estimation of covariation called the MI with the amino acid background distribution (MIB) is defined as:

Similarly, if *p*(*K^i^*, *L^j ^*) = 0, then the value of the monomial  is considered as 0.

In addition, we also normalize *MIB(K*, *L) *by *H(K*, *L) *and define a new measure MIB',

MIB' is sometimes referred to as the normalized MIB.

### The covariation of physicochemical properties

To measure the covariation of amino acid physicochemical properties, amino acids are grouped into ten overlapping groups as suggested by Taylor [37]. The ten overlapping groups are: hydrophobic (A, G, C, T, I, V, L, K, H, F, Y, W, M), aromatic (F, Y, W, H), aliphatic (I, V, L), tiny (A, S, G, C), small (P, N, D, T, C, A, G, S, V), proline (P), charged (K, H, R, D, E), negative (D, E), polar (N, Q, S, D, E, C, T, K, R, H, Y, W) and positive (K, H, R). In this section, we use *g_pair(a,b) *to denote the group pair, in which the former residue belongs to group *a *and the latter residue belongs to group *b*.

Then, the fractional frequency of group *a *in column *K *is defined as:

where count(*K*^*a*^) is the number of residues belongs to group *a *in column *K*.

The joint property distribution of *c_pair (K,L) *for *g_pair(a,b) *is defined as:

where the count(*K^a^*, *L^b^*) is the number of residue-pairs belonging to *g_pair(a,b)*.

We define a new measure of covariation as:

If the *p_p_*(*K^a^*, *L^b^*) = 0, the value of the monomial  is 0. If one column of the column-pair is fully conserved, the value is also 0. The measure is referred to as the MI of physicochemical properties (MIP) and the normalized MIP is defined as:

Where .

Moreover, amino acid physicochemical properties are also incorporated to the MIB model. The modified marginal fractional frequency of group *a *in column *K *is defined as:

Here, *q_p _*is the background distribution of physicochemical properties which is based on the *BLOSUM62 *amino acid background distribution.

Similarly, the covariation of the *c_pair(K,L) *is calculated as:

If *p_p_*(*K^a^*, *L^b^*) = 0, the value of the monomial  is considered as 0. The measure is referred to as the MIP with the physicochemical properties background distribution (MIBP) in the paper. And the normalized MIBP is defined as:

The measures MI', MIB', MIP' and MIBP' are used to quantify covariation in the paper.

### conn (k) is a more robust indicator than individual covariation score

It has been proved that individual MI values may be misleading [39]. In order to improve individual scores, [23] introduced an indicator *conn(k) *to characterize individual residues. In the paper, the top 75 high-scoring pairs are taken into account. And the cut-off of *conn(k) *is 5 in MI', MIB' and a comparing method ELSC. While in MIP' and MIBP', since there are ten groups, we take the top 25 high-scoring pairs and the cut-off of *conn(k) *is 3. Throughout this paper, we use *conn(k)*-name to denote the *conn(k) *score of the 'name' method. For example, *conn(k)*-MI' denotes the *conn(k) *score of MI'.

## Results and Discussion

**Figure 1:**
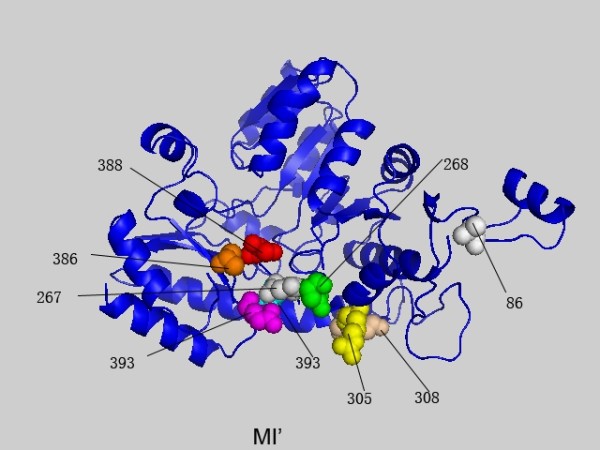
**The highest scoring residues k of 1QGN interlinked with position 388 based on MI'**. These sites are plotted in space filling mode and the site 388 is colored in red. Others are colored in turn of green, yellow, magenta, cyan, orange, tint white and grey with the decrease of the MI' values.

**Figure 2:**
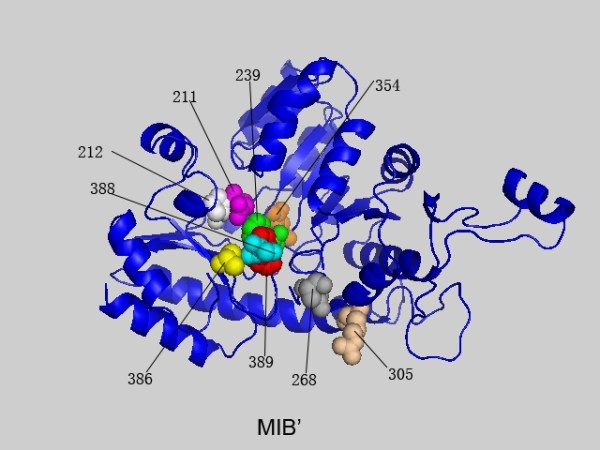
**The highest scoring residues k of 1QGN interlinked with position 389 based on MIB'**. These sites are plotted in space filling mode and the site 389 is colored in red. Others are colored in turn of green, yellow, magenta, cyan, orange, tint white and grey with the decrease of the MIB' values.

**Figure 3:**
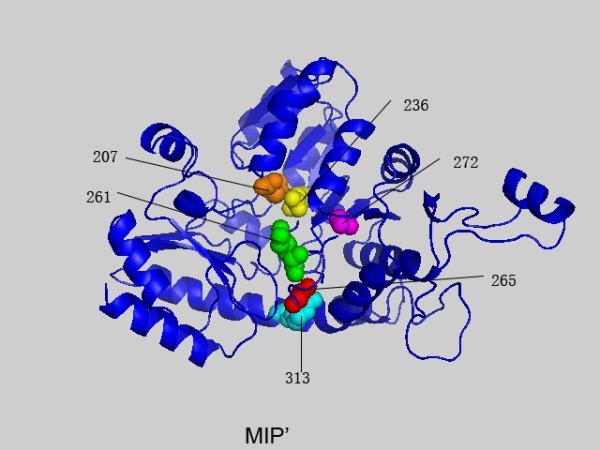
**The highest scoring residues k of 1QGN interlinked with position 265 based on MIP'**. These sites are plotted in space filling mode and the site 265 is colored in red. Others are colored in turn of green, yellow, magenta, cyan, orange, tint white and grey with the decrease of the MIP' values.

**Figure 4:**
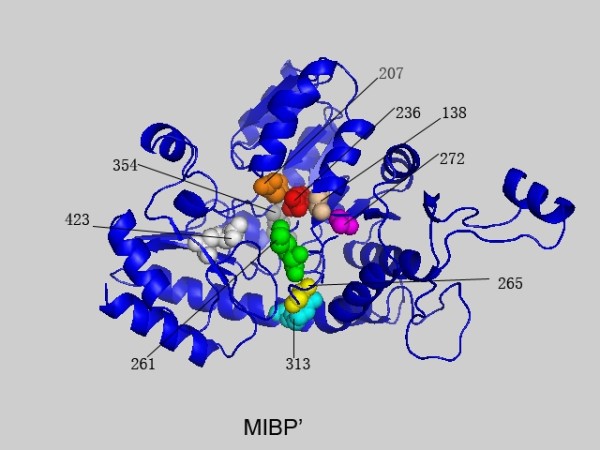
**The highest scoring residues k of 1QGN interlinked with position 236 based on MIBP'**. These sites are plotted in space filling mode and the site 236 is colored in red. Others are colored in turn of green, yellow, magenta, cyan, orange, tint white and grey with the decrease of the MIBP' values.

**Figure 5:**
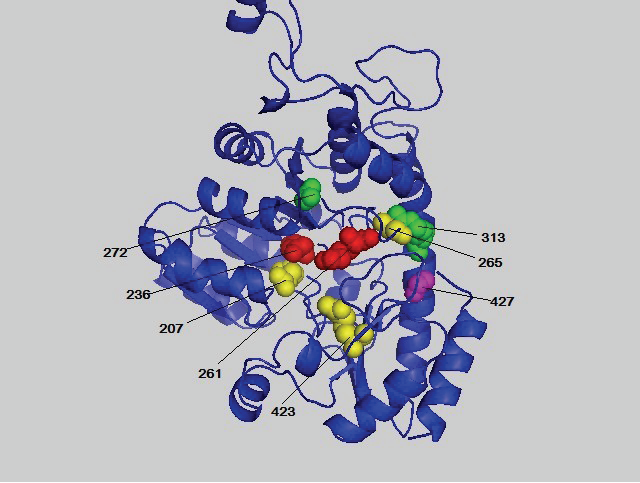
**The highest scored residues of 1QGN based on the conn(k)-MIBP' scores**. Residues of the 1QGN with *conn(k)*-MIBP' scores ≥4 are plotted in space filling mode and labeled. The sites 236 and 261 with the highest score 8 are colored in red. In addition, they are catalytic sites of the chain. Other chosen residues are colored in turn of green, yellow and magenta with the decrease of the *conn(k)*-MIBP' scores.

**Figure 6:**
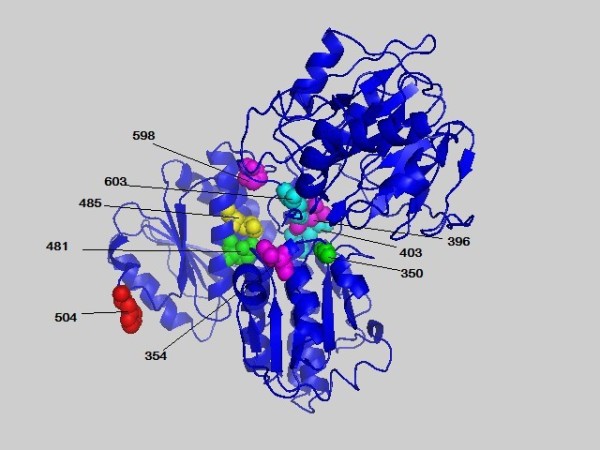
**The highest scored residues of 1JXA-A based on the conn(k)-MIBP' scores**. Residues of the 1JXA-A with *conn(k)*-MIBP' scores ≥3 are plotted in space filling mode and labeled. The site 504 with the highest score 8 is colored in red. Other chosen residues are colored in turn of green, yellow, magenta and cyan with the decrease of the *conn(k)*-MIBP' scores.

In order to compare our new methods with existing ones, all chosen methods, namely MI', *H2r*, ELSC, MIB', MIP' and MIBP' are tested on four MSAs. The first one is a toy MSA is shown in Table 1 with 6 sequences and each sequence has 6 residues. Others are commonly used protein families in comparing coevolution methods, which include 1JXA-A, 1B93-A [28] and PF01053 protein family. We use 1QGN to denote the related protein sequence of PF01053. Results on these MSAs are shown in Tables 2, 3, 4 and 5 and Figures 1, 2, 3, 4, 5 and 6[40]. However, ELSC is not applied to PF01053, for the method suffers an arithmetic overflow when sequence number is too large.

**Table 1:** A toy MSA used to illustrate the differences between these referred methods

C1	C2	C3	C4	C5	C6
D	A	W	A	E	E
D	A	W	A	E	F
D	A	W	A	E	D
D	A	Y	C	M	D
D	A	Y	C	M	T
D	A	Y	C	M	T

In the table, *Ci *denotes the *i*th column.

**Table 2:** The MI', MIB', MIP' MIBP' and ELSC scores based on the toy MSA

	MI'						MIB'					
		
	C1	C2	C3	C4	C5	C6	C1	C2	C3	C4	C5	C6
C1	0	0	0	0	0	0	0	0	0.138	0.237	0.141	0.003
C2		0	0	0	0	0		0	0.138	0.237	0.141	0.003
C3			0	1	1	0.296			0	1.375	1.279	0.360
C4				0	1	0.296				0	1.378	0.404
C5					0	0.296					0	0.362
C6						0						0

	**MIP'**						**MIBP'**					
		
	**C1**	**C2**	**C3**	**C4**	**C5**	**C6**	**C1**	**C2**	**C3**	**C4**	**C5**	**C6**

C1	0	0	0	0	0	0	0	33.425	36.015	29.586	3.655	5.364
C2		0	0	0	0	0		0	23.395	25.528	3.075	4.642
C3			0	0	0	0			0	27.707	3.387	5.030
C4				0	0.077	0.037				0	3.431	4.476
C5					0	0.053					0	1.854
C6						0						0

	**ELSC**											
		
	**C1**		**C2**		**C3**		**C4**	**C5**		**C6**		

C1	0		0		0		0	0		0		
C2			0		0		0	0		0		
C3					0		2.20	2.20		0.69		
C4							0	2.20		0.69		
C5								0		0.69		
C6										0		

In the table, *Ci *denotes the *i*th column.

**Table 3:** Comparisons of the H2r, MI', MIB', MIP' and MIBP' methods based on the PF01053 family

*H2r*		MI'		MIB'		MIP'		MIBP'	
***k***	***conn(k)***	***k***	***conn(k)***	***k***	***conn(k)***	***k***	***conn(k)***	***k***	***conn(k)***

388	10	388	10	389	30	265	5	236	8
305	6	272	8	388	9	236	5	261	8
276	6	313	8	393	8	261	5	313	6
307	5	207	7	391	8	313	4	272	6
389	5	138	7	268	7	423	4	423	5
285	4	393	6	386	7	272	4	265	5
386	4	236	6	305	7	427	4	207	5
389	4	268	6	280	6	207	4	427	4
		265	6	402	5				
		261	6	308	5				
		391	6	211	5				
		389	5						
		386	5						
		305	5						
		108	5						
		211	5						

In the table, *k *denotes the *k*th site of the 1QGN chain.

**Table 4:** Comparisons of the H2r, ELSC, MI', MIB', MIP' and MIBP' methods based on the 1JXA-A family

*H2r*		ELSC		MI'		MIB'		MIP'		MIBP'	
***k***	***conn(k)***	***k***	***conn(k)***	***k***	***conn(k)***	***k***	***conn(k)***	***k***	***conn(k)***	***k***	***conn(k)***

329	9	329	12	32	13	313	12	87	7	504	8
331	8	102	7	29	12	351	8	78	7	350	8
102	6	27	6	73	11	503	8	84	7	481	6
332	5	3	6	84	11	329	7	86	6	485	6
535	5	2	6	86	11	331	6	73	6	598	5
532	4	32	5	87	11	539	6	99	6	396	5
				78	11	502	6	29	4	354	5
				27	10	602	5	123	4	403	4
				123	10	254	5			603	3
				99	10	332	5				
				125	8						
				26	7						

In the table, *k *denotes the *k*th site of the 1JXA-A chain.

**Table 5:** Comparisons of the H2r, MI', MIB', MIP' and MIBP' methods based on the 1B93-A family

*H2r*		ELSC		MI'		MIB'		MIP'		MIBP'	
***k***	***conn(k)***	***k***	***conn(k)***	***k***	***conn(k)***	***k***	***conn(k)***	***k***	***conn(k)***	***k***	***conn(k)***

90	10	103	15	90	13	90	22	42	8	91	8
29	8	29	10	103	9	29	15	67	6	23	7
110	7	110	9	129	8	109	9	131	3	19	6
103	6	90	8	67	8	104	7	16	3	48	6
130	6	78	8	132	7	67	7			45	6
109	5	71	8	110	7	93	6			70	6
		99	8	71	7	97	6			69	6
		109	7	130	7	71	6			123	3
		104	6	131	6	103	5				
		89	6	29	6	110	5				
		67	6	104	6	99	5				
		97	5	109	5						
		65	5	65	5						

In the table, *k *denotes the *k*th site of the 1B93-A chain.

### Comparison of different methods on a toy MSA

In order to illustrate the effects of new biological constraints, we generate a toy MSA, which is shown in Table 1. In the MSA, column 1 and 2 are fully conserved which are used to illustrate the effect of the amino acid background distribution; column 3, 4 and 5 are used to illustrate the differences between the MIP' and MIBP' methods; and column 6 is randomly generated. We then ran and compared all the methods on the toy MSA, and the results are listed in Table 2. For convenience, a pair of columns *A *and *B *is represented by *c_pair(A,B) *in the study.

In this section, MI' and MIB' are compared to show the effect of incorporating amino acid background distribution. For column-pairs with one column fully conserved, such as *c_pair(1,3)*, *c_pair(1,4) *and *c_pair(1,5)*, the MI' scores are 0, while MIB' can distinguish them with different scores 0.138, 0.237 and 0.141, respectively. When the first column is fully conserved, the joint frequency of *c_pair(1,K) *is equal to the observed frequency of the residue represented by column *K*. On the other hand, the observed distribution of residues in column *K *is used as the marginal distribution, therefore the MI' score of *c_pair(1,K) *is 0. However for MIB', the marginal distribution is the observed distribution modified by the amino acid background distribution, thus, the MIB' scores of these column-pairs are distinguishable. For example, the MI' score of *c_pair(1,6) *is 0 while it is 0.003 for MIB'. Although the difference between 0 and 0.003 is not significant, it reveals that MI' and MIB' are essentially different. In general, the MI' scores of column-pairs with one fully conserved column are 0, while the MIB' method can distinguish them unless both columns are conserved. This is because that the marginal distribution used in the MIB' methods has been modified by the amino acid background distribution. In addition, the columns which are paired with the same column and get equal MI' scores, such as the *c_pair(3,6)*, *c_pair(4,6) *and *c_pair(5,6)*, are also distinguishable in MIB'. Although the distributions of column 3, 4 and 5 are the same in number, the type of amino acids are different. The result uncovers that the amino acid background distribution is meaningful in measuring the relationship between columns. For column-pairs with fully covariant columns, the MIB' scores might be different. For example the MIB' scores for *c_pair(3,4)*, *c_pair(3,5) *and *c_pair(4,5) *are 1.375, 1.279 and 1.378, respectively.

Different from methods based on amino acid frequencies, the MIP' measure is based on amino acids physicochemical properties. In detail, the MIP' values of *c_pair(4,5)*, *c_pair(4,6) *and *c_pair(5,6) *are 0.077, 0.037 and 0.053, respectively, while the MI' scores are 0.296. Table 2 shows that the covariation of physicochemical properties of *c_pair(4,5) *is stronger than that of *c_pair(4,6) *and *c_pair(5,6)*, while they are equivalent by MI'. Similar results can be found in the comparison of MIP' and MIBP'. Table 2 also shows that the MIP' score is 0 if one column in the pair is fully conserved while this scenario doesn't appear under the MIBP' measure. It worths noting that the score of *c_pair(1,2) *is 0 under the MI', MIB' and MIP' measures since these two columns are fully conserved. However, MIBP' can estimate the covariation of them and gives a score 33.425 to the column-pair. Another interesting observation is that: although column 3 is not fully conserved, the ten overlapping classifier can not distinguish W and Y. Thus the column with the fully conserved properties leads to a MIP' score of *c_pair(3,K) *0. So do G and A, I and L [30]. In contrast, under the MIBP' measure, these pairs can be distinguished due to the modification of marginal distribution by the background distribution of physicochemical properties.

### The effect of incorporating amino acid background distribution

In order to test the effect of incorporating amino acid background distribution, MIB' is compared with MI' and *H2r *on the PF01053 protein family. We plot the relatively high *conn(k)*-MIB' scored sites in Figure 5 and list the detailed information in Table 3. Table 3 shows that the differences among MIB', MI' and *H2r *are obvious. For example, the highest *conn(k)*-MIB' site is site 389, while that of MI' and *H2r *is site 388.

In addition, the number of the sites with *conn(k)*-MI'>= 5 is greater than that of MIB' and *H2r*. Moreover, site 389, a functional important site, gets the highest *conn(k)*-MIB', but its *conn(k)*-*H2r *value is less than 5. It is already known that in the models of the substrate tCGS (cystathionine *γ*-synthase from *Nicotiana tabacum*) complex (1QGN) [41], the movement of O3' from a mainly hydrophobic environment, arounds site 389 (Phe). Furthermore, sites 239 (Phe) and 211 (Asn), which are essential for maintaining functional environment, are ranked as the first and third sites interlinked with site 389 by the MIB' method. As shown in Figure 2, sites, which get have relatively high MIB' scores with site 389, form a network around it. It demonstrates that strongly covariation sites surround important functional domains to make a compensatory effect to maintain the function of the protein, a conjecture being validated by many case studies [5,13,39]. These case studies are also tested on the 1B93-A family and the general results are similar.

Besides *H2r *and MI', MIB' was also compared with the ELSC method proposed by [26]. ELSC uses a perturbation-based algorithm for calculating explicit likelihood of subset covariance, however the background distribution is also ignored. ELSC and MI' are similar since the background distribution is not taken into account for both methods. As shown in Table 4, the sites with high *conn(k)*-MIB' and those of ELSC are different. Only site 329 is predicted by both methods. Besides, the sites with high *conn(k)*-MIB' scores are mostly in the N terminal while that of ELSC are dispersed. On the 1B93-A family the ELSC shows no significant advantage over other methods. Moreover, it suffers arithmetic overflow when sequence number of the MSA is too large. In general, these results suggest that incorporating amino acid background is very important in measuring coevolution.

### Coevolution of amino acid physicochemical properties

In contrast to previous methods which consider amino acids as symbols in a uniformly diverse alphabet, MIP' and MIBP' try to account for amino acid physicochemical properties. As shown in Table 3, the sites predicted by MIP' and MIBP' are different to those by MI' and MIB'. The highest conn(k)-MI' site is site 388, while that of MIP' and MIBP' is site 265 and 236, respectively. In addition, some sites predicted by MIP' and MIBP' are also predicted by MI', but not by MIB'. These results demonstrate that the classification of amino acids and physicochemical properties are different in depicting the MSA. Moreover, site 236 and 261 in PF01053 family, site 504, 481, 485 and 603 in 1JXA-A family, and site 19 and 91 in 1B93-A family are catalytic residues. It means that there are physicochemical properties based networks to maintain the catalytic environment or support the catalytic process.

An in-depth analysis on PF01053 family is also given to show the performances of MIBP'. It has been proven that the carboxylate OD2 of site 236 stabilizes the positively charged pyridine nitrogen of PLP [41]. Site 261 takes its role in catalysis by tCGS named ping-pong mechanism in the first and final steps. They coevolve to maintain specific physicochemical environment stabilization in or around the active region. As shown in Table 4, the high *conn(k)*-MIBP' scored sites are in the N-terminal of the sequence while that of the MIP' are in the C-terminal. It demonstrates that the results are affected by background distribution significantly. Moreover, four of five catalytic residues are highly scored in the 1JXA-A family and the related structure [42]. Catalytic sites are located in the N-terminal half of the domain at the carboxyl edge of the *β*-sheet. And the sites with high *conn(k)*-MIBP' scores are in the isomerase domain of GlmS (248-608) which is responsible for the binding of Fru6P and its conversion to GlcN6P. However site 504 is separated to other high scoring sites in space. In [43], it was shown that residue 504 plays a key role in the sugar ring opening on a different polypeptide chain. A Schiff base with residue 603 is formed and it is replaced by the incoming ammonia in the Schiff base. It also indicates that the mutation of residue 603 from Lys to Arg results in a decreasing of the synthase activity and an increasing of the isomerase activity. Site 485 forms H bond with the hydroxyl groups of the sugar.

To compare with the conservation information, the JSD method [28] is used to rank the degree of sites in the 1JXA-A family at different identity thresholds and the related results are shown in the supplement material. As shown in Table 6, the sites with high *conn(k) *scores are not highly scored by the JSD. Among the sites predicted by MI', only sites 86 and 84 are in the top ten of the JSD rank. And in the MIB', the sites are less conserved than MI'. Moreover, for MIP' and MIBP', although the sites are relatively conserved, the JSD rank can not correspond to the *conn(k) *value. In general, the relevance of conservation and covariation is not so high, which demonstrates that the conservation and covariation information are relatively independent properties of proteins.

**Table 6:** Comparison of conservation method and coevolution methods based on 1JXA-A family

*conn(k)*-MI' rank	JSD rank	*conn(k)*-MIB' rank	JSD rank	*conn(k)*-MIP' rank	JSD rank	*conn(k)*-MIBP' rank	JSD rank
32	64	313	310	87	12	504	2
29	23	351	28	78	59	350	52
73	21	503	42	84	10	481	29
84	10	329	97	86	3	485	27
86	3	331	288	73	21	598	11
87	12	539	24	99	56	396	35
78	59	502	134	29	23	354	16
27	65	602	73	123	19	403	26
123	19	254	45			603	32
99	56	332	151				
125	36						
26	31						

The column of *conn(k)*-name rank represents the sites with *conn(k) *scores in 'name' method. And the sites are presented from the high *conn(k) *scores to low ones. The column of JSD rank represents the corresponding JSD rank of the site.

### Relations between different measures

A comparison of the MI', MIB', MIP' and MIBP' measures was given based on the MSA of PF01053 and the related protein structure is PDB ID: 1QGN[28]. In Figures 1, 2, 3 and 4 we found that with different biological constraints, the highest scoring sites are different and the comutating sites are also different. In the MI' method, the highest scoring site is 388 while that of MIB', MIP' and MIBP' are 389, 265 and 236, respectively. On the other hand, to make certain differences between these measures, we used the Friedman test to judge whether the performance statistics for these measures are significantly different. The difference between the performances of two measures is called statistically significant if the *P*-value estimated by the Friedman test with Bonferroni correction is less than 0.05 [28].

To testify the validity of the amino acid background distribution, the MI' and MIB' measures were compared. As shown in Table 7, the *P*-value between MI' and MIB' is 6.82 × 10^-4^, thus there is significant difference between them. It confirmed the conclusion of [25] that the biological constraints is meaningful in measuring covariation, and evolution pressures of amino acids are removed successfully by using their background distribution. To clarify differences between covariation of amino acids and covariation of amino acid physicochemical properties, the MI' and MIP' measures were also compared. The *P*-value between the MI' and MIP' measures is 2.03 × 10^-7^. The *P*-value indicates that the covariation of amino acids is significantly different to that of physicochemical properties. These results indicate that the covariation of physicochemical properties is a new aspect of biological information to detect coevolution.

**Table 7:** The P-values between the MI', MIB', MIP', MIBP' methods

Measure	MI'	MIB'	MIP'	MIBP'
MI'	1	6.82 × 10^-4^	2.03 × 10^-7^	7.7 × 10^-2^
MIB'		1	9.63 × 10^-8^	3.68 × 10^-8^
MIP'			1	8.27 × 10^-1^
MIBP'				1

### Effect of sequence identity threshold

The effect of sequence identity thresholds in measuring coevolution is tested in this section. Sequence identity thresholds of 90%, 80%, 70% and 60% are chosen as examples on the 1JXA-A protein family. The identity thresholds < 60% are ignored since the account of the number of sequences is less than125. Performances of MIB' on the protein family are shown in Table 8 and performances of other methods are shown in the supplement materials. Table 8 shows that sites change significantly with the decreasing of the identity thresholds and the numbers of the predicted sites are also decreasing. For example, site 313 is the highest *conn(k)*-MIB' scoring site at the identity thresholds of 90%, 80% and 70%, while it is not predicted at the identity threshold of 60%. And at relatively high identity thresholds, site 238 is not predicted while its *conn(k)*-MIB' score is high at relatively low identity thresholds. Moreover, there are only 3 sites left at the identity threshold 60%. It means these three sites coevolve with many columns which do not coevolve with other columns strongly. While at 90% threshold, the information is enough to reveal the covariation of those columns. Thus, it demonstrates that the lower identity threshold affects the covariation significantly. It may lead to the consequence that some sites without coevolved property are mistakenly regard as the highly coevolved sites. In contrast, some sites with coevolved property are not predicted since the signal of coevolution reduces with the lower identity. It is true that sequences with high identity would reduce the correctness of the MSA, thus identity threshold of 95% or 90% is acceptable. The related results are submitted with the paper.

**Table 8:** Performances of MIB' at different sequence identity

90		80		70		60	
***k***	***conn(k)***	***k***	***conn(k)***	***k***	***conn(k)***	***k***	***conn(k)***

313	12	313	16	313	12	238	24
351	8	400	6	238	11	332	8
503	8	332	6	502	8	331	7
329	7	502	6	331	6		
331	6	329	6	332	5		
539	6			329	5		
502	6						
602	5						
254	5						
332	5						

The MIB' method is tested on the 1JXA-A protein family at sequence identities 90%, 80%, 70% and 60%.

## Conclusions

In this study, we propose two new biological constraints, based on which several new measures are designed to detect protein residue coevolution. The first constraint is amino acid background distribution which is used to develop the MIB' method. In contrast to previous methods which focused on how to transform joint count to joint frequency, MIB' method tries to remove the effects of amino acids evolutional pressures in the measure. By incorporating the new biological constraints, we found that MIB' is more effective in measuring amino acid coevolution. The second constraint is the physicochemical properties of amino acids which are used in the MIP' method. Motivated by the MIB' method, MIBP' method which removes the physicochemical properties' evolutional pressures is also proposed. Results show that the MIBP' method is sensible to catalytic sites. It indicates that physicochemical properties of residues around catalytic sites are strongly evolved. Moreover, results show that the MIBP' measure is significantly different from methods based on amino acid distribution. Thus the covariation of physicochemical properties supplies new coevolution information.

## Authors' contributions

HG carried out the studies on MIB, MIP and MIBP, performed the sequence alignment and drafted the manuscript. YD designed the methods to this study, performed the statistical analysis and helped to draft the manuscript. JY and JW participated in the discussion, and helped to draft the manuscript. All authors have read and approved the final manuscript.

## Supplementary Material

Additional file 1 **Data sets**. All MSAs used in the study are given in the file 'Additional-File-1.zip'. Click here for file

Additional file 2 **Results**. A zip file (Addtional-File-2.zip) contains all results on above data sets. Click here for file

Additional file 3 **Perl Code**. A zip file (Perl-codes.zip) contains all Perl codes used in the study. Click here for file

Additional file 4 **Effects of sequence identity**. The supplement material includes the comparisons of these proposed methods on different sequence identities based on the 1JXA-A family. Their corresponding JSD ranks are also shown in the material. Click here for file
