# Supplementary material for: New methods to measure residues coevolution in proteins
Source: BMC Bioinformatics. 2011 May 26;12:206. doi: 10.1186/1471-2105-12-206 (PMC3123609; doi:10.1186/1471-2105-12-206)
Supplement: Additional file 4 — Effects of sequence identity. The supplement material includes the comparisons of these proposed methods on different sequence identities based on the 1JXA-A family. Their corresponding JSD ranks are also shown in the material. [file 1471-2105-12-206-S4.PDF]

Supplement Material

Table 1 - Comparisons of the MI', MIB', MIP' and MIBP' methods based at the 1JXA-A on identity 80%

In the table, k denotes the kth site of the 1JXA-A chain.

MI'		MIB'		MIP'		MIBP'	
k	$conn(k)$	k	$conn(k)$	k	$conn(k)$	k	$conn(k)$
29	11	313	16	86	7	504	8
32	10	332	6	87	6	481	8
73	9	400	6	73	6	375	7
84	9	502	6	99	6	403	6
87	9	329	6	78	6	349	4
78	9			84	6	351	5
27	7			29	5	350	4
86	7					354	4
123	7						
99	7						
603	6						
601	5						
1	5						

Table 2 - Comparisons of the MI', MIB', MIP' and MIBP' methods based at the 1JXA-A at identity 70%

In the table, k denotes the kth site of the 1JXA-A chain.

MI'		MIB'		MIP'		MIBP'	
k	$conn(k)$	k	$conn(k)$	k	$conn(k)$	k	$conn(k)$
29	10	313	12	84	5	375	8
73	10	238	11	87	5	481	6
87	10	502	8	73	4	403	6
78	10	331	6	86	4	504	5
84	10	332	5	29	4	351	4
27	9	329	5	78	4	354	4
26	9						
32	9						
99	8						
1	7						
505	6						
123	6						
504	6						
596	5						
603	5						
601	5						
86	5						
598	5						

Table 3 - Comparisons of the MI', MIB', MIP' and MIBP' methods based on the 1JXA-A at identity 60%

In the table, k denotes the kth site of the 1JXA-A chain.

MI'		MIB'		MIP'		MIBP'	
k	$conn(k)$	k	$conn(k)$	k	$conn(k)$	k	$conn(k)$
26	9	238	24	26	7	481	11
87	9	332	8	29	7	399	10
29	9	331	7	32	7	191	5
99	9			87	6		
73	8			73	6		
27	8			27	6		
84	8			78	6		
78	8			84	5		
32	8						
598	7						
485	7						
504	7						
603	7						
596	7						
601	7						
505	7						
606	7						

Table 4 - JSD rank of the sites with high conn(k) scores in the MI', MIB', MIP' and MIBP' methods based on the 1JXA-A at identity 80%

The column of *conn(k)*-name rank represents the sites with *conn(k)* scores in 'name' method. And the sites are presented from the high *conn(k)* scores to low ones. The column of JSD rank represents the corresponding JSD rank of the site.

conn(k)-MI' rank	JSD rank	*conn(k)*-MIB' rank	JSD rank	*conn(k)*-MIP' rank	JSD rank	*conn(k)*-MIBP' rank	JSD rank
29	21	313	314	86	3	504	1
32	61	332	164	87	12	481	26
73	19	400	88	73	19	375	8
84	10	502	138	99	54	403	24
87	12	329	96	78	57	349	23
78	57			84	10	351	22
27	62			29	21	350	49
86	3					354	13
123	18						
99	54						
603	29						
601	77						
1	4						

Table 5 - JSD rank of the sites with high conn(k) scores in the MI', MIB', MIP' and MIBP' methods based at the 1JXA-A on identity 70%

The column of *conn(k)*-name rank represents the sites with *conn(k)* scores in 'name' method. And the sites are presented from the high *conn(k)* scores to low ones. The column of JSD rank represents the corresponding JSD rank of the site.

conn(k)-MI' rank	JSD rank	*conn(k)*-MIB' rank	JSD rank	*conn(k)*-MIP' rank	JSD rank	*conn(k)*-MIBP' rank	JSD rank
29	25	313	322	84	10	375	8
73	16	238	407	87	11	481	21
87	11	502	141	73	16	403	26
78	56	331	328	86	4	504	1
84	10	332	191	29	25	351	17
27	58	329	93	78	56	354	12
26	24						
32	64						
99	54						
1	5						
505	49						
123	15						
504	1						
596	14						
603	22						
601	63						
86	4						
598	9						

Table 6 - JSD rank of the sites with high conn(k) scores in the MI', MIB', MIP' and MIBP' methods based at the 1JXA-A on identity 60%

The column of *conn(k)*-name rank represents the sites with *conn(k)* scores in 'name' method. And the sites are presented from the high *conn(k)* scores to low ones. The column of JSD rank represents the corresponding JSD rank of the site.

conn(k)-MI' rank	JSD rank	*conn(k)*-MIB' rank	JSD rank	*conn(k)*-MIP' rank	JSD rank	*conn(k)*-MIBP' rank	JSD rank
26	23	238	358	26	23	481	19
87	14	332	243	29	27	399	43
29	27	331	367	32	58	191	25
99	49			87	14		
73	22			73	22		
27	60			27	60		
84	11			78	59		
78	59			84	11		
32	58						
598	9						
485	24						
504	1						
603	20						
596	13						
601	53						
505	44						
606	18						

Table 7 - JSD rank of the sites with high conn(k) scores in the MI', MIB', MIP' and MIBP' methods based on the 1B93-A

The column of *conn(k)*-name rank represents the sites with *conn(k)* scores in 'name' method. And the sites are presented from the high *conn(k)* scores to low ones. The column of JSD rank represents the corresponding JSD rank of the site.

conn(k)-MI' rank	JSD rank	*conn(k)*-MIB' rank	JSD rank	*conn(k)*-MIP' rank	JSD rank	*conn(k)*-MIBP' rank	JSD rank
90	56	90	56	42	50	91	6
103	84	29	17	67	22	23	5
129	91	109	38	131	138	19	1
67	22	104	60	16	25	48	3
132	94	67	22			45	7
110	100	93	61			70	10
71	26	97	48			69	9
130	123	71	26			123	13
131	138	103	84				
29	17	110	100				
104	60	99	30				
109	38						
65	32						
